# Identification of Phase-Separation-Protein-Related Function Based on Gene Ontology by Using Machine Learning Methods

**DOI:** 10.3390/life13061306

**Published:** 2023-05-31

**Authors:** Qinglan Ma, FeiMing Huang, Wei Guo, KaiYan Feng, Tao Huang, Yudong Cai

**Affiliations:** 1 School of Life Sciences, Shanghai University, Shanghai 200444, China; mql1117@shu.edu.cn (Q.M.); hfm123@shu.edu.cn (F.H.); 2 Key Laboratory of Stem Cell Biology, Shanghai Jiao Tong University School of Medicine (SJTUSM) & Shanghai Institutes for Biological Sciences (SIBS), Chinese Academy of Sciences (CAS), Shanghai 200030, China; gw_1992@sjtu.edu.cn; 3 Department of Computer Science, Guangdong AIB Polytechnic College, Guangzhou 510507, China; kyfeng@gdaib.edu.cn; 4 Bio-Med Big Data Center, CAS Key Laboratory of Computational Biology, Shanghai Institute of Nutrition and Health, University of Chinese Academy of Sciences, Chinese Academy of Sciences, Shanghai 200031, China; 5 CAS Key Laboratory of Tissue Microenvironment and Tumor, Shanghai Institute of Nutrition and Health, University of Chinese Academy of Sciences, Chinese Academy of Sciences, Shanghai 200031, China

**Keywords:** phase-separation protein, gene ontology, machine learning

## Abstract

Phase-separation proteins (PSPs) are a class of proteins that play a role in the process of liquid–liquid phase separation, which is a mechanism that mediates the formation of membranelle compartments in cells. Identifying phase separation proteins and their associated function could provide insights into cellular biology and the development of diseases, such as neurodegenerative diseases and cancer. Here, PSPs and non-PSPs that have been experimentally validated in earlier studies were gathered as positive and negative samples. Each protein’s corresponding Gene Ontology (GO) terms were extracted and used to create a 24,907-dimensional binary vector. The purpose was to extract essential GO terms that can describe essential functions of PSPs and build efficient classifiers to identify PSPs with these GO terms at the same time. To this end, the incremental feature selection computational framework and an integrated feature analysis scheme, containing categorical boosting, least absolute shrinkage and selection operator, light gradient-boosting machine, extreme gradient boosting, and permutation feature importance, were used to build efficient classifiers and identify GO terms with classification-related importance. A set of random forest (RF) classifiers with F1 scores over 0.960 were established to distinguish PSPs from non-PSPs. A number of GO terms that are crucial for distinguishing between PSPs and non-PSPs were found, including GO:0003723, which is related to a biological process involving RNA binding; GO:0016020, which is related to membrane formation; and GO:0045202, which is related to the function of synapses. This study offered recommendations for future research aimed at determining the functional roles of PSPs in cellular processes by developing efficient RF classifiers and identifying the representative GO terms related to PSPs.

## 1. Introduction

Numerous organelles or compartments are contained in eukaryotic cells. In addition to well-known membrane-bound organelles, such as the Golgi apparatus, endoplasmic reticulum (ER), and mitochondria, membrane-less organelles are crucial for specifying and compartmentalizing distinct cellular processes in eukaryotic cells [1]. Nucleoli, Cajal bodies, promyelocytic leukemia bodies, processing bodies (P-bodies), and stress granules (SGs) are only a few examples of the numerous membrane-less organelles. They are present in the nucleus and the cytoplasm [2,3].

Membrane-less organelles could be assembled by liquid–liquid phase separation (LLPS), which is a transient supramolecular condensation of different proteins, nucleic acids, and other biomolecules [4,5]. The low-complexity domains and intrinsically disordered regions (IDRs) of the protein promote weak multivalent interactions to make a key contribution to LLPS [4,6,7,8]. Post-translational modifications of proteins, especially protein phosphorylation, also promote LLPS [9]. LLPS compartments are thought to facilitate the interaction of cellular components, such as proteins and RNA, or to keep them away from undesired reactions [4,6]. Many cellular metabolic processes are known to be regulated by LLPS, and abnormal LLPS leads to the development of metabolic diseases, such as type 2 diabetes mellitus, Alzheimer’s disease, and metabolic bone disease [10]. Therefore, clarifying the mechanism of uncontrolled LLPS development is crucial to prevent pathogenic transformation.

The proteins involved in LLPS are called phase-separation proteins (PSPs). Hemoglobin, for example, has been shown to experience phase separation at high concentrations in vitro [11,12]. However, phase separation in living cells is only expected to occur in a small number of proteins with particular sequence-dependent properties [13]. Under stress conditions, RNA-binding proteins and RNA undergo phase separation, mediating the formation of SGs in the cytoplasm to inhibit translation in vivo [14,15]. Although the LLPS of biomolecules has been intensively studied in recent years, knowledge of PSPs is still lacking.

Machine learning is an effective technique for predicting PSP. Representative PSP prediction tools, such as PScore [16], LARKS [17], PLAAC [18], Fuzdrop [19], and PSPredictor [20], aid in screening PSPs. PLAAC is based on prion-like structural domains, PScore is based on the expected number of long-range planar sp^2^ pi-pi contacts, and LARKS is based on low-complexity aromatic-rich kinked chain segments. However, while all first-generation PSP predictor methods were based on small samples and specific features [21], the newly introduced FuzDrop and PSPredictor were trained with much larger samples, with PSPredictor achieving a ten-fold cross-validation accuracy of 94.71% on an external test set. Above methods were effective to predict PSP. However, they cannot fully uncover essential differences between PSPs and non-PSPs. The essential biomarkers that can be used to directly identify PSPs from non-PSPs have not been fully investigated.

In this study, an investigation on PSPs and non-PSPs was conducted from a different point of view. It is known that Gene Ontology (GO) is widely used in bioinformatics, which is a type of annotation information indicating the essential properties of proteins. The identification of highly related GO terms of PSPs is helpful for us to understand underlying mechanism of PSPs, and at the same time, these GO terms can be used to distinguish PSPs from non-PSPs. Previous studies have not investigated PSPs from the point of view on GO terms. In view of this, each PSP or non-PSP was encoded according to their GO annotation information using one-hot scheme. Then, multiple machine learning methods were employed to analyze such a big dataset. In detail, five feature ranking algorithms, including categorical boosting (CATboost) [22], extreme gradient boosting (XGBoost) [23], least absolute shrinkage and selection operator (LASSO) [24], light gradient-boosting machine (LightGBM) [25], and permutation feature importance (PFI) [26,27], were adopted to sort GO features, generating five feature lists. These lists were fed into incremental feature selection (IFS) [28], incorporating random forest (RF) [26] as the classification algorithm, to extract essential GO features and build efficient classifiers. Some GO features were analyzed and can be confirmed to be related to validated functions of PSPs. On the basis of the representative GO features of PSPs reported in this study, future studies of PSP function can be conducted.

## 2. Materials and Methods

### 2.1. Data

This study used experimentally validated PSPs and non-PSPs derived from PhaSePred [29]. The phase separation-self and -part proteins from Chen et al. were considered positive samples in this study, with a total of 588 PSPs [29]. Meanwhile, 59,857 non-PSPs offered by Chen et al. were used as negative samples [29]. As PSPs and non-PSPs were encoded by their GO annotation information, those without such information were discarded. As a result, 578 PSPs and 58,563 non-PSPs were obtained and investigated in this study. These proteins are provided in Table S1. Sequences of these proteins were obtained from UniPort. The GOA database was used to retrieve the GO terms for each investigated protein, yielding a total of 24,907 GO terms. Based on these GO terms, each protein was encoded into a 24,907-dimensional binary vector. Given a protein, if it was annotated by a GO term, the corresponding component in the vector for that term was marked as 1; otherwise, it was marked as 0.

### 2.2. Feature Ranking Algorithms

To date, lots of GO terms have been designed to annotate proteins. In this study, more than 24,000 GO terms were involved. Evidently, only a part of them is highly related to PSPs. These GO terms can be identified by advanced computational methods. However, a single method can only discover a few essential GO terms as each method has limitations. In view of this, this study employed five feature ranking algorithms, including CATboost [22], XGBoost [22], LASSO [24], LightGBM [25], and PFI [26,27]. These algorithms were designed using quite different principles, meaning that they can overview the dataset from different points of view. Accordingly, a full discovery can be accessed based on them. Their brief descriptions are as follows.

#### 2.2.1. Categorical Boosting

CATboost is an open-source gradient-boosting machine-learning algorithm used for solving classification and regression problems [22]. The importance of a feature could be computed by the contribution it makes in building the trees; the more it is used and the more it affects the predictions, the more important it is. Here, Prediction Value Change was used to estimate the importance of each feature. The values of a feature are randomly permuted several times. Each time, the change in the prediction outputs is calculated. The change in the *i*th permutation is denoted as $k_{i}$ and the importance of the feature is calculated as the average change over all permutations, $FI=\sum_{i=0}^{n}k_{i}$. All features are sorted in accordance with their importance in descending order.

#### 2.2.2. Extreme Gradient Boosting

XGBoost is an open-source software library for gradient boosting, a machine-learning technique used to produce accurate models for supervised learning problems [23]. XGBoost calculates feature importance to help identify the most significant features that contribute to the prediction. As for a single tree $f_{i}$, the importance of a feature r, denoted as $I(r,f_{i})$, is estimated by its information gain in splitting the internal nodes, weighted by the number of the samples in the internal node. If a feature has not been used in a tree, its importance is set to 0. The importance of a feature r in the whole model is measured by averaging the importance values of all trees, which is computed as $I\left(r\right)=\frac{1}{t}\sum_{i=1}^{t}I(r,f_{i})$, where t is the total number of the trees. Then, all features could be ranked by their importance values.

#### 2.2.3. Least Absolute Shrinkage and Selection Operator

LASSO is a linear regression technique that adds a penalty term to the cost function to reduce the magnitude of the coefficients of some features to zero [24]. In LASSO, the magnitude of the coefficients implicitly determines the importance of the feature, with features with larger absolute coefficients being seen as more significant. Features that have coefficients that are almost 0 are viewed as being less significant and may be eliminated from the model.

#### 2.2.4. Light Gradient-Boosting Machine

LightGBM is a gradient-boosting framework that addresses supervised learning issues by employing tree-based learning techniques [25]. LightGBM calculates feature importance in several manners: (1) Split: the number of times a feature is used in a split; (2) Gain: the average gain of the feature when used in a split; (3) Coverage: the average coverage of the feature, defined as the number of samples affected by the splits that use the feature. Here, the setting of split was used as a metric in measuring the importance of features.

#### 2.2.5. Permutation Feature Importance

Permutation feature importance operates by permuting a single feature’s values at random and observing the effect on the model’s performance [26,27]. The values of a single feature are randomly permuted to calculate the permutation feature importance, and the model is re-trained and re-evaluated. This process is repeated for each feature in the dataset, and the average performance degradation for each feature could be used as a measure of its importance.

The above five algorithms were applied to the PSPs and non-PSPs that were represented by GO features. Each algorithm outputs a feature list. For easy descriptions, the five lists were called CATboost, XGBoost, LASSO, LightGBM, and PFI feature lists, respectively.

### 2.3. Incremental Feature Selection

To determine which part of features in each above-mentioned feature list were essential for PSPs, the IFS method was employed in this study. It is a classic feature selection method that iteratively adds features from a list, with the goal of identifying the optimal subset of features that provides good prediction accuracy [28,30,31]. IFS starts with an empty set of features and iteratively adds features that results in the greatest improvement in the F1 score. The process continues until all features have been fed into the classifier. This process always employs a step for adding features when a huge number of features are involved. Here, a 10-step interval was used to add the features from each feature list. When adding ten features, current features and the target variables were combined to feed into a classification algorithm for building the classifier. The classifier was evaluated by 10-fold cross-validations [32]. After all classifiers have been evaluated, the best classifiers, measured by F1 score in this study, can be obtained. Features used in this classifier were deemed to be essential, which constitute the optimal feature subset.

### 2.4. Synthetic Minority Oversampling Technique

According to Section 2.1, non-PSPs were much more than PSPs. Thus, the dataset was extremely imbalanced. The classifier built on such dataset may produce bias and its evaluation results may not be reliable. Thus, we employed synthetic minority oversampling technique (SMOTE) [33] to tackle this problem. SMOTE is a popular data augmentation method for handling class imbalance in supervised learning problems. This method creates synthetic samples for the minority class to correct the imbalance between classes. The synthetic samples are created by interpolating between two samples from the minority class that were chosen at random. The original dataset is supplemented with synthetic samples, thereby growing the minority class and balancing the distribution of the classes. The SMOTE algorithm in this study was implemented via Python, which can be obtained at https://github.com/scikitlearn-contrib/imbalanced-learn (accessed on 23 March 2023).

### 2.5. Random Forest

RF is an ensemble machine learning algorithm that uses decision trees to make predictions [26,34,35,36,37,38]. It combines the results of various decision trees to create a consensus prediction to lower variance and boost the overall model accuracy. Each tree in an RF is trained by using a randomly chosen subset of the data and a randomly chosen collection of features for each split. By reducing the correlation between different trees and preventing overfitting, this randomization creates a more reliable and accurate model.

### 2.6. Performance Evaluation

The F1 score, also known as the F1 measure [39,40,41], is a common metric used in classification tasks, particularly in binary classification problems. It is a measure of the accuracy of a model in correctly predicting the positive class, while also minimizing false positives and false negatives.

The F1 score is calculated as the harmonic mean of precision and recall. Precision measures the proportion of true positive predictions among all positive predictions, while recall measures the proportion of true positive predictions among all actual positive samples. The F1 score combines both precision and recall, giving equal weight to both metrics, and is calculated as follows:

(1) $$F1\;score=\frac{2\times (precision\times recall)}{\left(precision+recall\right)}$$

## 3. Results

**Figure 1:**
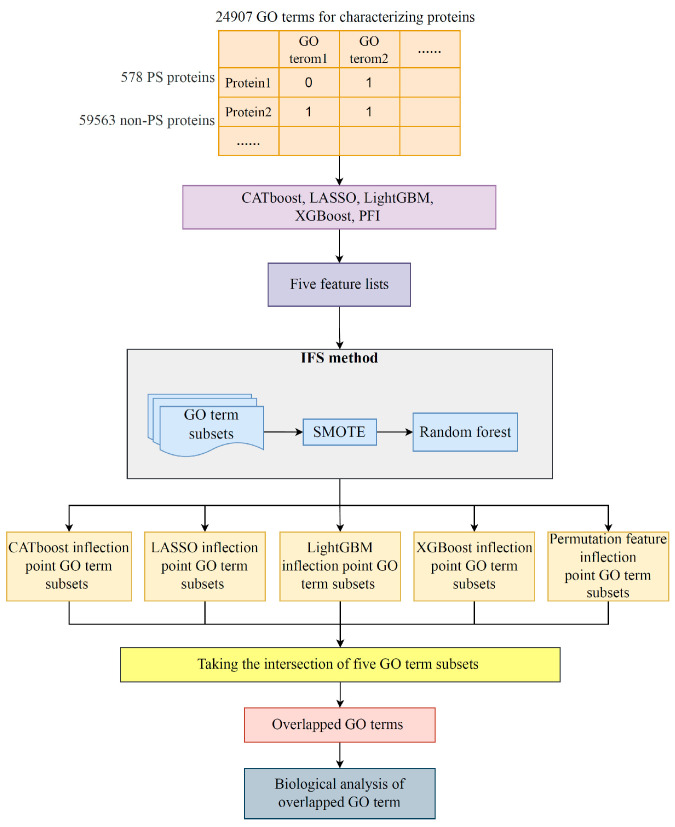
Flowchart of the machine learning procedure that integrates multiple feature ranking algorithms and IFS computational framework. A total of 578 PS proteins and 58,563 non-PS proteins were collected from the public database and treated as positive and negative samples, respectively. A total of 24,907 GO terms were extracted to characterize the proteins. Subsequently, five feature ranking algorithms, namely, CATboost, LASSO, LightGBM, XGBoost, and PFI, were used to evaluate the importance of different GO terms for classification. Each sorted GO term list was divided into different subsets and inputted to the IFS framework to obtain the optimal feature subset, the inflection point feature subset, and the optimal classifier. The five inflection point feature subsets are intersected to obtain the overlapped GO terms, and the overlapped GO terms were biologically analyzed.

In this study, advanced feature importance assessment methods and an IFS framework were combined to mine GO terms that facilitate the distinction between PSPs and non-PSPs. Figure 1 displays the overall computational architecture. Below is a description of the outcomes connected to each stage of the computation process.

### 3.1. Feature Ranking Results

The importance of the 24,907 GO terms used to describe PSPs and non-PSPs varies for differentiating PSPs and non-PSPs. CATboost, LASSO, LightGBM, XGBoost, and PFI were performed to assess how different GO terms contribute to the classification in this case. In Table S2, the GO terms are sorted from highest to lowest importance to the classification in five feature lists, named CATboost, LASSO, LightGBM, XGBoost, and PFI feature lists, respectively. Different feature ranking algorithms have different principles and assumptions and therefore provide different perspectives on the importance of GO terms. This could be useful in providing a more comprehensive understanding of the feature importance and the underlying relationships in the data.

### 3.2. Results of IFS Method with Random Forest

**Figure 2:**
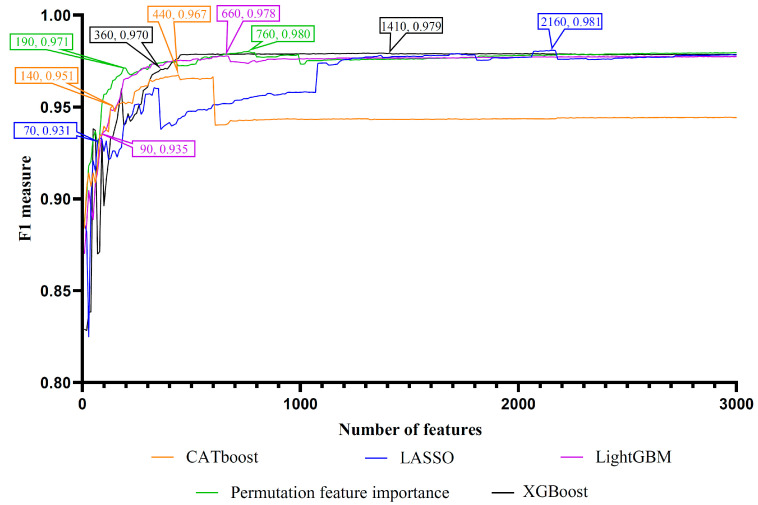
Incremental feature selection (IFS) curves of random forest on five feature lists yielded by five feature ranking algorithms. Five IFS curves were used for the CATboost, LASSO, LightGBM, XGBoost, and PFI feature lists. On each IFS curve, the number of features and F1 measure corresponding to the optimal GO term subset and the inflection point GO term subsets were indicated.

Lots of features were included in each feature list. If all features were considered in the IFS method, it would cost lots of time. On the other hand, only a few features were highly related to the identification of PSPs. Thus, we only considered the top 3000 features (GO terms) in each feature list. These features in each list were divided in intervals of ten to produce different sizes of feature subsets. An RF classifier was built on each feature subset and evaluated by 10-fold cross-validation. The detailed results are shown in Table S3. To clearly show the performance of RF classifiers under different feature subsets, an IFS curve was plotted for each feature list with the F1 score acting as the y-axis and the number of features acting as the x-axis, as shown in Figure 2.

The IFS results showed that the optimal classification performance for RF was achieved when the top 440 (CATboost feature list), 2160 (LASSO feature list), 660 (LightGBM feature list), 1410 (XGBoost feature list), and 760 (PFI feature list) features were selected from the corresponding feature list. The F1 scores of these RF classifiers were 0.967, 0.981, 0.978, 0.979, and 0.980, respectively. Accordingly, the optimal feature subsets were obtained from five feature lists. The detailed performance, including recall, precision and F1 score, of these RF classifiers is provided in Table 1. Among them, the RF classifier using the top 2160 features in the LASSO feature list achieved the highest F1 score of 0.981. A notable detail that all above RF classifiers based on different feature lists had F1 scores over 0.960. These efficient RF classifiers could be used to distinguish PSPs from non-PSPs.

### 3.3. Intersection of Most Essential Features Extracted from Different Feature Lists

**Figure 3:**
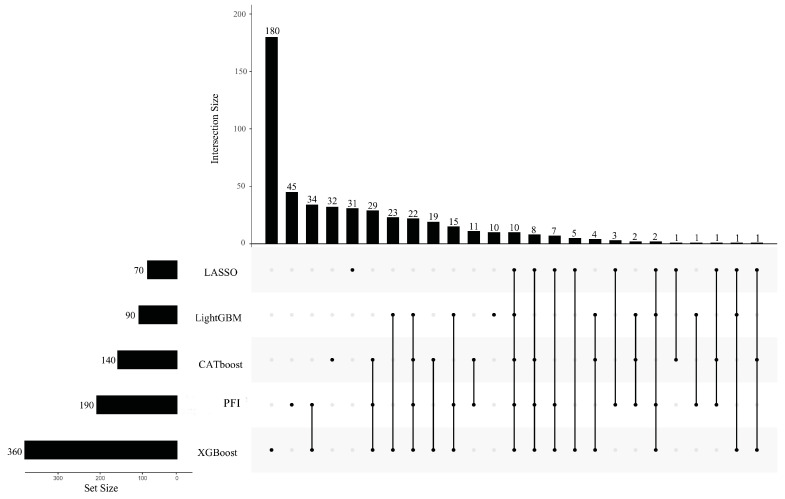
Upset graph to show the intersection of five inflection feature subsets identified from five feature lists that were created by five feature ranking algorithms. The GO terms identified by multiple feature ranking algorithms indicated that they were more likely to differ in PSPs and non-PSPs.

As mentioned in Section 3.2, five optimal feature subsets were obtained from five feature lists. Features in these subsets may be essential for describing PSPs. However, too many such features were involved. The smallest subset contained 440 features. The union of these subsets included 3608 features. It is difficult to give a detailed analysis of so many features. Thus, the extraction of most essential features from these features was necessary. For the IFS results on one feature list (Table S3), we can discover a feature subset that contained much less features than the optimal feature subset, whereas the corresponding RF classifier gave a little lower performance than the best RF classifier. By careful checking, the top 140 (CATboos feature list), 70 (LASSO feature list), 90 (LightGBM feature list), 360 (XGBoost feature list), and 190 (PFI feature list) features in the corresponding feature list can be used to constitute the feature subsets satisfying the above requirements. These subsets were called CATboost, LASSO, LightGBM, XGBoost, and PFI inflection feature subsets. The corresponding inflection points on five IFS curves are marked in Figure 2, alone with the F1 score of the RF classifier using these features. The detailed performance of RF classifiers with inflection feature subsets is listed in Table 1. It can be observed that these classifiers used much less features and provided a little lower performance than the best classifiers on the same feature list. Thus, we can confirm that these features were most crucial to the identification of PSPs. The union of five inflection feature subsets contained 497 features. Evidently, some features can belong to more than one subset, indicating that they were identified by multiple feature ranking algorithms. Thus, the intersection of the inflection feature subsets was taken, and an upset graph was drawn, as shown in Figure 3. The features (GO terms) belonging to 1–5 subsets are provided in Table S4. Ten features were in all inflection feature subsets, indicating that they were identified to be most essential by all five feature ranking algorithms. The discussion section of this paper focuses on the biological significance of the GO terms identified by multiple feature ranking algorithms.

## 4. Discussion

In this study, a set of potential GO features was identified by the computational approach, revealing the partial function of the PSPs. These identified protein GO features could help identify and understand PSPs. According to some recent publications, the identified protein GO features are closely related to important functions in which PSPs are known to be involved.

### 4.1. Phase Separation in RNA Binding-Related Biological Process

The formation of many membrane-less organelles is mediated by LLPS of key proteins and nucleic acid scaffolds, including P-bodies, SGs, and nucleoli [4]. The computational approach in the present work identified several RNA binding-related protein GO features, including RNA binding (GO:0003723), mRNA binding (GO:0003729), and nucleic acid binding (GO:0003676). Moreover, some GO features, such as regulation of mRNA stability (GO:0043488), positive regulation of transcription by RNA polymerase II (GO:0045944), RNA polymerase II-specific DNA-binding transcription factor binding (GO:0061629), regulation of transcription by RNA polymerase II (GO:0006357), and regulation of DNA-templated transcription (GO:0006355), are directly associated with transcription and translation.

Ribonucleoprotein (RNP) is a conjugate of RNA-binding protein (RBP) and RNA. It is widely considered to be a membrane-less organelle induced by LLPS for its generation [42]. On the basis of the fact that the RBPs involved in the formation of RNP undergo phase separation, the predicted GO features of RNA-binding-associated proteins were therefore justified.

P-bodies and SGs are two membrane-free organelles that occur in the cytoplasm as a result of RNA and protein phase separation [43], which are cytoplasmic RNP granules. P-bodies and SG-associated protein GO features were identified by the computational approach, including cytoplasmic stress granule (GO:0010494) and P-body (GO:0000932). They are involved in post-transcriptional regulation and translational control [44]. P-bodies were discovered to consist mainly of translationally repressed mRNAs and proteins linked to mRNA degradation, indicating a potential function in post-transcriptional control [45]. According to research on the component enrichment of mRNA in organelles, P-bodies are crucial regulators of major biological processes, such as chromatin regulation and RNA processing [46]. Depending on the function of P-bodies, the RNA binding-associated GO features, and transcription-associated GO features mentioned above may also help predict PSPs. SGs assemble when cells are subjected to external stress, serving as a protective mechanism [44,47]. The assembly of SGs was found to be dependent on an RNA network composed of core proteins, such as G3BP1 [48,49]. G3BP1 could sense the concentration of free RNA in the cell and binds to it, and the conformational change leads to phase separation [50]. According to the function of the PSP G3BP1 in SGs, the RNA binding-related GO features mentioned may be important during phase separation in SGs.

Assembly of the nucleolus is thought to be LLPS-driven [51,52]. Moreover, many nucleolar proteins have IDRs that are essential for LLPS driving [52]. Therefore, the identified SP protein feature, nucleolus (GO:0005730), could be used as a valid feature. Nucleolus is the site of ribonucleoprotein particle assembly, and its main function is to synthesize ribosomes [53]. In the nucleolus, rDNA transcription, rRNA processing, and rRNA ribosomal protein assembly could be distinguished by LLPS [54]. RNA polymerase I (RNA Pol I) is known to be an abundant protein in the nucleolus, and its function is to participate in the transcription of rRNA [52]. Thus, depending on the function of the nucleolus, proteins that phase separate in the nucleolus may have RNA binding and transcriptional features that were identified in this study.

### 4.2. Phase Separation in Membrane Formation

Protein phase separation could disassemble membrane-bound organelles into condensates, mediate condensate transport across membrane-bound organelles, and participate in the assembly of adhesion complexes and signaling clusters on the plasma membrane [55]. The computational approach in the present study identified a number of GO features associated with cell membranes and membrane-bound organelles, such as membranes (GO:0016020), plasma membrane (GO:0005886), and intracellular membrane-bounded organelle (GO:0043231).

Phase separation of some proteins occurs at the plasma membrane. Phase separation is caused by multivalent interactions between plasma membrane proteins and the cytoplasm, and adhesion complexes and signaling clusters seem to form as a result [55,56,57]. Therefore, the predicted PSP features of signal transduction (GO:0007165) and cadherin binding (GO:0045296), in addition to the plasma membrane-related features, are reasonable. Moreover, tight junctions between epithelial or endothelial cells could be formed by proteins on the plasma membrane via LLPS [55]. The cellular scaffolding protein ZO was found to play a key role in the phase separation of tight junctions, driven by multivalent interactions through its conserved PDZ-SH3-GuK superstructure domain [58,59]. In addition, ZO proteins inhibit the phase separation process by phosphorylation [59]. This evidence partially validates cytoskeleton (GO:0005856), protein kinase binding (GO:0019901), and protein phosphorylation (GO:0006468).

The ER is where some proteins undergo phase separation, involving ER membrane (GO:0005789). TIS granules are functional regions on the ER containing mRNA encoding membrane proteins, with phase-separated assembly mediated by the RNA-binding protein TIS11B [60,61]. Thus, the PSPs in TIS granules should have mRNA binding and the membrane-bound organelle binding function mentioned before. In addition, LLPS may potentially be used for the construction of autophagosome nucleation sites, commonly known as omegasomes, on the ER substructure domain [55,62,63]. ATG proteins are recruited for autophagosome formation [64,65], playing a role in the different steps of autophagosome formation. The prediction of hydrolase activity (GO:0016787), autophagy (GO:0006914), and protein binding (GO:0005515) of PSPS in the present study may be relevant.

### 4.3. Phase Separation at the Synapse

LLPS may form presynaptic knob and postsynaptic density (PSD) of neuronal synapses [66]. Therefore, synapse (GO:0045202), the GO feature predicted for the PSP, is the valid feature. Each synaptic site has a tightly packed, protein-rich compartment known as the PSD, which is in charge of receipt, amplification, and storage of signals started by the presynaptic cell [67,68]. The presynaptic active zone, an electron-dense region below the plasma membrane in the presynaptic compartment, controls the rate and magnitude of neurotransmitter release [69].

Phase separation could be used to create PSD assemblies [66], thus validating the PSD (GO:0014069) identified in the present study. Initial observation revealed a possible phase separation between PSD-95 and SynGAP [70]. The recombinant PSD system demonstrated that AMPA receptor regulatory proteins (TARPs) could be aggregated into PSD by phase separation, and TARP-PSD-95 multivalent interaction is essential for synaptic transmission [71]. Moreover, site-specific phosphorylation of PSD-95 was found to dynamically regulate PSD through phase separation [72].

The presynaptic active zone is also thought to undergo protein phase separation [73], thus validating the structural constituent of presynaptic active zone (GO:0098882) identified in the present study. RIMs and RIM-BPs are important active zone proteins for anchoring readily releasable pool SVs to fusion sites [74]. By using in vitro recombinant techniques, purified RIM and RIM-BP mixtures were found to undergo phase separation at physiological protein concentrations [75]. In addition, the core active-zone scaffolding proteins SYD-2 (also known as lipoprotein-α) and ELKS-1 were found to undergo phase separation during the early stages of synaptic development, mediating the assembly of the synaptic active zone [73].

## 5. Conclusions

In this study, PSPs that underwent experimental validation and real non-PSPs were gathered as positive and negative samples, respectively. Every protein was encoded into a binary vector according to its GO terms. Some essential GO terms were identified by investigating such classification problems. Ten GO terms that were identified to be most essential by all five ranking algorithms are thought to be highly relevant to distinguishing PSPs from non-PSPs. A number of GO terms, such as GO:0003723, GO:0016020, and GO:0045202, were discussed in terms of their significance in distinguishing PSPs. At the same time, a number of RF classifiers were built using the IFS framework on the basis of feature subsets extracted from different feature lists generated by five feature ranking algorithms. The best RF classifier had an F1 score of 0.981. By developing efficient RF classifiers and identifying representative GO terms related to PSPs, this work contributes to an enhanced understanding of the molecular mechanisms that regulate phase separation and the role of membranelle compartments in cellular processes.

## Figures and Tables

**Table 1:** Performance of key random forest classifiers on each feature list.

Feature List	Number of Features	Recall	Precision	F1 Score
CATBoost feature list	440	0.942	0.995	0.967
	140	0.910	0.995	0.951
LASSO feature list	2160	0.968	0.994	0.981
	70	0.875	0.996	0.931
LightGBM feature list	660	0.962	0.994	0.978
	90	0.882	0.995	0.935
XGBoost feature list	1410	0.964	0.995	0.979
	360	0.947	0.995	0.970
PFI feature list	760	0.967	0.994	0.980
	190	0.949	0.995	0.971

## Data Availability

The data presented in this study are openly available in PhaSePred, reference number [29].

## Supplementary Materials

The following supporting information can be downloaded at: https://www.mdpi.com/article/10.3390/life13061306/s1, Table S1: Phase-separation proteins and non-phase-separation proteins. Table S2: List of features (GO terms) sorted by CATboost, LASSO, LightGBM, XGBoost, and PFI methods; Table S3: IFS results of random forest with multiple evaluation metrics for each feature list; Table S4: Intersection results for inflection feature subsets extracted from CATboost, LASSO, LightGBM, XGBoost, and PFI feature lists.
